# Case of Diseased Brain, Complicated with Extraordinary Effusion of Fluid into the Ventricles, and Separation of the Cranial Bones

**Published:** 1816-10

**Authors:** Shirley Palmer


*Case of diseased Brain, complicated with extraordinary Effusion of Fluid into the Ventricles, and Separation of the Cranial Bones.* By SHIRLEY PALMER, M.D.

HISTORY. Among the poor who resort to my house, on stated mornings, for gratuitous advice, a fine and interesting girl of six years, was, in August 1815, presented by her mother. There was a something in her general manner and appearance, which strongly attracted my attention. About one year previously to this period, she had suffered rather severely from measles; and her constitution, till then, displaying all the characters of robust health, sustained a shock from which it never afterwards recovered. A few doses of senna infusion were administered by the parents; but the health of the child continued slowly and progressively to decline. She now presented the ordinary train of symptoms by which confirmed disorder of the bowels is signalized:—flushed cheeks, tum'd upper lip, furred tongue, swollen abdomen without induration, hot skin, rapid and irritable pulse. Precarious appetite, frequent rejection of food by vomiting, great irritation about the nostrils, disturbed sleep, fæces irregular, both as to the period of evacuation, colour, and consistence, and scanty and turbid urine, were other signs reported by the mother; whose extraordinary solicitude for the child constitutes some pledge of the vigilance, and perhaps correctness, of her observation. But general pain of the head, with vertigo and indistinct vision, was the symptom of which the child principally, and almost constantly, complained. The pupil of the eye, larger than natural, contracted sluggishly on the stimulus of light; and the whole organ exhibited an appearance strongly resembling that which I have noted in incipient amaurosis. A calm but deep expression of internal suffering dwelt upon her countenance, and formed a striking contrast with the intelligent smile by which it was, now and then, lighted up. No external violence had, at any time, been inflicted on the cranium. The integuments, covering it, possessed their natural temperature. There was no sensible increase of force or fulness in the stroke of the carotid, temporal, or occipital arteries. The physiognomy of the child was calculated to awaken suspicions of a scrophulous taint; but no instance of her suffering from glandular enlargement, abscess, or cutaneous affection, could be recollected. The health of her parents was generally good, their habits regular.

A vigorous course of mercurial purgatives and light regimen were prescribed, and steadily followed up for some weeks without decided benefit; for, although the functions of the abdominal viscera were more regularly executed, the intestinal secretions improved, and the vomiting suppressed, no favourable change had taken place in the condition of the brain and optic nerve. On the contrary, the vertigo, the dimness and obscurity of sight, were rather aggravated. The inference was obvious: either the affection of the brain must have been, with respect to the abdominal disorder, the pre-existent malady; or, if secondary, have become, from duration, independent of the cause by which it had been originally excited; and, of course, no longer curable by the destruction of such cause. These opinions, and the painful conclusion to which they lead, were unreservedly explained to the mother; and, just as I was meditating a more energetic plan of treatment for the child's relief, her attendance was abruptly discontinued: nor would any distinct recollection of these circumstances have probably been preserved, but that the interesting appearance and hopeless condition of the little invalid, had made an impression on my mind not readily to be obliterated.

On Saturday, July 13, I accidentally heard of the child's death, about midnight of the preceding Thursday, with uncommon enlargement of the head, and after an illness of long and extraordinary suffering. This was an opportunity of indulging my insatiable thirst for morbid dissections, not to be neglected. I immediately applied to Mr. Bird, Junior, Surgeon of this place, who had watched the progress of the fatal malady to its close, and left nothing untried which science or humanity could suggest for its mitigation. This gentleman not only very obligingly allowed me to assist him in the examination of the body, but communicated every particular respecting the principal phenomenæ and treatment of the disease, necessary to complete its history.

Shortly after the child had ceased, from aggravated illness, to attend at my house, a chronic abscess, without evident cause, formed on the great toe of the right foot. In course of time, it brake, and discharged copiously; and, although the purulent secretion from the wound was suppressed, or nearly so, for six weeks before death, the orifice never again completely closed. The regularity of the bowels, which the plan of purgation had served to re-establish, was no more conspicuously disturbed, except that their excretions acquired, towards the close of life, a blackish appearance and fœtid odour. Day after day, all the signs of the cerebral affection put on a more marked and portentous character. Some time before Christmas, the cranium was discovered to possess an increase of volume; and, about the commencement of the present year, the functions of the optic nerve were so greatly impaired, that the glare of a candle held to the eye was scarcely visible. The head, meanwhile, continued to enlarge; and the pains which generally, and particularly towards the occipital region, affected it, were constant and distressing. At length, the child became incapable of supporting the erect posture. For three months, she lay in total darkness, and passed her excrements involuntarily. The pulse was weak and frequent; the urinary secretion scanty. She had no abdominal pains, no strabismus, no partial or general convulsion of the muscular system. Her memory continued remarkably clear; and all her senses, except that of vision, perfectly unimpaired to the last. The principal remedies employed by Mr. Bird were preparations of quicksilver and digitalis, internally; counter-irritation externally, by successive blisters to the occiput.

*Morbid appearances.* On Sunday morning, July 14, fifty-seven hours after death, the body was examined by Mr. Bird and myself. Upon *external view*, it was greatly emaciated; the abdominal muscles sunk in and greenish, and the face so completely changed, that I could recognize in it no trace of the sweet and intelligent countenance which, a year before, had so deeply interested me. The cranium equalled, in volume, a large adult skull. Its circumference, taken at the superior transverse occipital ridge, posteriorly, and half an inch above the supraciliary ridges of the frontal bone, anteriorly, measured twenty-two inches and a quarter; its length, from the occipital tubercle to the union of the nasal bones with the frontal, fifteen inches; the distance from the superior margin of one zygoma to the corresponding point of the other, fourteen inches and a half.* All the cranial sutures displayed a separation of from half to three-fourths of an inch. There was the relic of a scrofulous ulcer of the great toe, probably complicated with caries.

*Encephalon.* While the bones were submitted to the action of the saw, about one ounce of yellow thickish fluid gushed from the right ear. On separating the calvaria,† the dura mater presented itself, exceedingly tense and beautifully injected. Even the more minute ramifications of the three meningeal arteries were distinctly visible. The superior longitudinal sinus, and the inferior, as it afterwards appeared, were empty. The whole brain evidently formed a capacious sac, with thin parietes, containing fluid. A distinct sense of fluctuation was communicated to the finger, on touching it. Separation of the hemispheres exposed the corpus callosum, not half an inch below the surface, thin, transparent, unusually broad, and agitated by the undulations of the subjacent fluid. While we were observing this appearance, a sudden gush took place from the posterior part of the brain. Much of the fluid consequently was lost, ere a vessel could be reached to receive it. By an horizontal section of the right hemisphere, the corresponding lateral ventricle was opened about half an inch below the surface. It was enormously dilated, and nearly full of fluid. The corpus striatum and thalamus nervi optici had not their wonted convexity and distinctness of aspect. The septum lucidum was, at least, an inch in depth, exceedingly delicate. No accumulation of fluid was perceptible in the fifth ventricle. The foramen

---

* From accurate measurement of three subjects, I have found the mean dimensions of the *healthy female cranium about the seventh year*, to stand thus:—Circumference, inches $19\frac{7}{8}$; length, $12\frac{1}{2}$; breadth, 12. Hence the increase of volume in the present case is at once evident.

† In this instance, I first tried the plan of opening the cranium proposed by Professor Chaussier, and twice described in this Journal See vol. i. page 135, and vol. ii. page 147). It exceeded my most sanguine expectations; for although, from the thinness and almost disunited state of the cranial bones, the difficulties of separation were unusually great. I was, by this mean, enabled to effect my purpose without the slightest injury of the dura mater. Henceforth, a trephine will constitute an essential part of my dissecting apparatus.

of Monro would have admitted the extremity of my little finger. The plexus choroides was of a pale obscurely red colour, wasted, knotty, and so soft as to lacerate on the slightest touch. The reflected and posterior horns of the ventricle shared the dilatation. The pes hippocampi and hippocamus minor were less distinctly prominent than usual. The left lateral ventricle presented similar appearances, but was somewhat less capacious than its fellow. The fornix was so soft, that, in an attempt to reflect it, the structure was utterly destroyed. Of the parts situated posteriorly, the third ventricle, velum interpositum, tubercula quadrigemina, and pineal gland, no distinct traces remained: they were involved in an indiscriminate mass of morbid pulp. With some difficulty, the fourth ventricle was made out: it would have admitted an adult thumb. The substance of the brain was so softened as to defy every attempt at clean dissection; and the whole of the great cavity formed by the dilated ventricles, was lined with a matter resembling fresh cheese-curd, which continually adhered to the scalpel and fingers, and much impeded my progress. In the cerebral substance, constituting the parietes of this cavity, and especially about the natural situation of the pineal gland, were imbedded several small, indurated bodies, precisely resembling the pulmonary tubercle of scrophulous subjects. On attempting to invert the whole brain for inspection of its base, the posterior left lobe was found strongly adherent to the tentorium cerebelli, and to the dura mater investing that portion of the temporal and occipital bones: it was converted into a yellow, firm, compact mass, without any vestige of original structure. A woman in attendance very aptly compared it to "soaked bread" in a state of compression. Great difficulty was encountered in tearing this mass from its containing cavity. The cerebellum, although little changed in appearance, was unnaturally soft. Our view of the basis of the brain was rendered exceedingly confused and indistinct, by the state of the parts and difficulty of removal. All the nerves, of cerebral origin, particularly those of the seventh, eighth, and ninth pairs, were very thin, as though from a wasting of their substance. The second pair was flattened so as strikingly to resemble in figure the olfactory. The medulla oblongata was quite sound. The sinuses in the basis cranii were uncommonly turgid with blood. The quantity of fluid caught during the dissection was about one pound. Appearances on the table and floor bespoke an effusion of more than half that quantity. The whole may be safely calculated at twenty-five ounces.* It was of a faint straw colour, and slightly turbid.

*Thorax.* Examination of this and the abdominal cavity was greatly disturbed and interrupted by the violent and unwelcome intrusion of one of the child's relatives. The lungs were free from adhesion, and sound, as were the bronchial glands. The right sac of the pleura contained about ten drachms of serous fluid; the left less than an ounce. The heart, in situ, felt uncommonly hard and firm. The pericardium contained about half an ounce of limpid serum. The pulmonary cavities of the organ were in a natural state. The ventricle displayed, near the origin of the pulmonary artery, a mass of adherent fibrine. The parietes of the aortic ventricle, and its columnæ carneæ, possessed unusual strength. The former had a degree of thickness more considerable than in the healthy adult heart; but the cavity was not dilated. The aortic valves were more strong and rigid than natural; and from their base, a considerable distance up the aorta, this vessel exhibited an appearance, as if small but clearly defined pieces of cartilage were inlaid, here and there, in the internal membrane. Traces of chronic inflammation were visible between. The pink blush of the latter, contrasted with the whiteness of the cartilage, produced an effect singular as beautiful. The coronary arteries, unusually large, were otherwise sound.

*Abdomen.* Few morbid appearances were presented here. The ileum was inflamed for about two feet of its length. In some parts, the intestine was quite livid, as though disorganization were impending. The vessels of the mesentery were gorged with black blood; and the whole membrane forming it, seemed to participate the inflammation of the ileum. The mesenteric glands were, in general, large as a horse-bean, but in a state of flaccidity rather than induration. The liver was quite sound, and the gall-bladder distended with healthy bile.

*Reflections.* From a retrospect of the history and mor-

---

* A small hydrocele trocar might, I conceive, be advantageously employed to draw off the fluid from the brain, whenever the accumulation was so large as to menace rupture of the containing cyst. Thus the whole fluid might be received into a basin, and its quantity be correctly ascertained.

bid appearances of the case just detailed, the following conclusions may, I think, be legitimately drawn:

First. The malady which destroyed the child was decidedly scrophulous. Such inference is alike supported by the indolent abscess of the toe, and the unequivocal character of the morbid structure of the brain: it receives additional support from the circumstance of the invasion of the disease having immediately succeeded the decline of measles.

Secondly. The brain was the original seat of the morbid action, and the iliac lesion a secondary or consequent affection. This is proved by the comparatively more advanced and confirmed state of the cerebral, with respect to the intestinal malady, and by the regularly progressive aggravation of all the symptoms characterizing the former, even when the abdominal disturbance had signally yielded to the agency of medicine. The enormous accumulation of fluid in the ventricles of the brain was probably determined by the irritation, morbid or mechanical, of the indurated posterior lobe.* Hence the case differs essentially from hydrencephalus in its more common and acute form; where the cephalic effusion is *generally* the consequence of irritative or inflammatory action, propagated from some portion of the intestinal tube to the brain.

Thirdly. In the chronic nature of the cerebral disease, inducing only the process of *gradual* effusion, and in the early age of the patient, admitting considerable separation of the cranial bones, may be found a satisfactory explication of some of the more striking phænomena which the case presented: the progressive enlargement of the head; the accommodation of the brain to the increasing pressure of the central fluid, and consequent preservation of its functions. Yet that the memory, the senses (the power of vision excepted), and all the more delicate affections and propensities of the human mind, should survive, unimpaired, the general wreck or compression of that portion of the cerebral organ, universally allowed to constitute the "throne of intellect," is one of those facts which most decidedly militate against the fashionable but evanescent doctrines of the German craniologists.

Fourthly. Decided thickening had taken place in the parietes of the left ventricle of the heart. Whether it

---

* Is it probable that the posterior lobe could exercise a pressure on the torcular Herophili and left lateral sinus, capable of inducing, or aggravating, the cerebral effusion? I am inclined to think not.

arose from obstruction in the circulation of the carotid and vertebral arteries, or from increased resistance of the rigid aortic valves, is, perhaps, doubtful: but had it even been possible to cure the cerebral lesion, the left ventricle would unquestionably have formed, in its turn, the focus of morbid action—the seat of incurable organic disease.

Lastly. It may be observed, that enlargement of the cranium, from an internal cause, does not *invariably* depend on effusion of fluid. The brain, like all other glandular organs, may acquire an unnatural volume from simple increase of its solid structure. This is a fact, perhaps not generally understood. I have recently met with a case, in a celebrated German Journal, which clearly illustrates it; and which I intend to transcribe for the Foreign Department of this, or our following Number.

*Tamworth, August,* 1816.

---

